# A type III effector protease NleC from enteropathogenic *Escherichia coli* targets NF-κB for degradation

**DOI:** 10.1111/j.1365-2958.2011.07568.x

**Published:** 2011-02-22

**Authors:** Jaclyn S Pearson, Patrice Riedmaier, Olivier Marchès, Gad Frankel, Elizabeth L Hartland

**Affiliations:** 1 Department of Microbiology and Immunology, University of MelbourneMelbourne, Vic. 3010, Australia.; 2 Centre for Immunology and Infectious Disease, Blizard Institute of Cell and Molecular Science, Barts and The London School of Medicine and Dentistry, Queen Mary University of LondonLondon E1 2AT, UK.; 3 Centre for Molecular Microbiology and Infection, Division of Cell and Molecular Biology, Imperial College LondonLondon SW7 2AZ, UK.

## Abstract

Many bacterial pathogens utilize a type III secretion system (T3SS) to inject virulence effector proteins into host cells during infection. Previously, we found that enteropathogenic *Escherichia coli* (EPEC) uses the type III effector, NleE, to block the inflammatory response by inhibiting IκB degradation and nuclear translocation of the p65 subunit of NF-κB. Here we screened further effectors with unknown function for their capacity to prevent p65 nuclear translocation. We observed that ectopic expression of GFP–NleC in HeLa cells led to the degradation of p65. Delivery of NleC by the T3SS of EPEC also induced degradation of p65 in infected cells as well as other NF-κB components, c-Rel and p50. Recombinant His^6^-NleC induced p65 and p50 cleavage in HeLa cell lysates and mutation of a consensus zinc metalloprotease motif, HEIIH, abrogated NleC proteolytic activity. NleC inhibited IL-8 production during prolonged EPEC infection of HeLa cells in a protease activity-dependent manner. A double *nleE/nleC* mutant was further impaired for its ability to inhibit IL-8 secretion than either a single *nleE* or a single *nleC* mutant. We conclude that NleC is a type III effector protease that degrades NF-κB thereby contributing the arsenal of bacterial effectors that inhibit innate immune activation.

## Introduction

Bacterial pathogens such as enteropathogenic *Escherichia coli* (EPEC) and enterohaemorrhagic *E. coli* (EHEC) utilize a type III secretion system (T3SS) to translocate multiple effector proteins into infected cells (Coburn *et al*., 2007). The T3SS of EPEC and EHEC is encoded within the locus of enterocyte effacement (LEE) pathogenicity island which is essential for the ability of the pathogens to cause attaching and effacing (A/E) lesions (McDaniel and Kaper, 1997). A/E lesions are characterized by intimate attachment of the bacteria to the host cell surface and the recruitment of filamentous actin beneath the adherent bacteria (Frankel *et al*., 1998). Intimate attachment results from a high-affinity interaction between the outer membrane adhesin, intimin and its receptor, Tir, which is translocated into the host cell membrane by the LEE-encoded T3SS (Kenny *et al*., 1997; Hartland *et al*., 1999; Luo *et al*., 2000).

Apart from A/E lesion formation, effectors translocated by the LEE encoded T3SS mediate a range of pathophysiological events including the disruption of cellular tight junctions, inhibition of apoptosis and the suppression of innate immune signalling pathways. Recent advances in the study of EPEC/EHEC effector biology have revealed important new biochemical activities for many effectors with previously unknown function. For example, NleH is a potent inhibitor of apoptosis that acts by binding Bax inhibitor 1 (Hemrajani *et al*., 2010). Other effectors with newly ascribed functions in promoting cell survival and cell attachment are EspO and EspZ. EspO and its homologue, OspE, in *Shigella* promote host cell attachment to the basement membrane by interacting with integrin-linked kinase (ILK) and blocking the turnover of focal adhesions (Kim *et al*., 2009). EspZ also prevents host cell detachment by interacting with the host protein CD98 and enhancing the stability of focal adhesions during infection (Shames *et al*., 2010). The NleG type III effectors are a newly described family of U-Box E3 ubiquitin ligases. Although their various targets are unknown, the NleG proteins are likely to direct the turnover of host cell proteins and/or other type III effectors by the cell proteasome through the host cell ubiquitination pathway (Wu *et al*., 2010). Other EPEC/EHEC effectors interfere with host cell Rho GTPase activity. EspT is a rare WxxxE effector that confers an invasive ability on EPEC by activating Rac1 and Cdc42, thereby stimulating membrane ruffling and lamellipodia formation (Bulgin *et al*., 2009). EspM2 is another WxxxE effector that acts as a RhoA guanine nucleotide exchange factor (Arbeloa *et al*., 2010). EspH has a seemingly opposing activity that involves binding the DH-PH domain of multiple RhoGEFs, thereby preventing Rho nucleotide exchange and enzyme activation (Dong *et al*., 2010). How the overlapping and sometimes opposing activities of these effectors are co-ordinated during infection remains to be discovered.

The transcription factor NF-κB is a key regulator of cytokine gene expression. In most tissues, the dominant form of NF-κB comprises a dimer of the subunits p65 and p50. However other NF-κB proteins can also form homo- and heterodimers including c-Rel, RelB and p52. Together these factors are termed Rel proteins as they all share an N-terminal Rel homology domain which is involved in dimerization. In resting cells, the p65/p50 dimer is held in an inactive state by binding the inhibitor, IκB. Upon stimulation by exogenous stimuli such as Toll-like receptor (TLR) ligands or cytokines such as TNF and IL-1β, IκB is phosphorylated, ubiquitinated and then degraded by the cell proteasome (Li and Verma, 2002). The release of p65/p50 from IκB allows the dimer to translocate through the nuclear pore complex to the cell nucleus where p65 stimulates gene expression from NF-κB-dependent promoters such as *IL8* (Li and Verma, 2002). Recently, we and another research group reported that the type III effectors, NleE and NleB from EPEC, blocked activation of NF-κB by inhibiting IκB degradation and p65 nuclear translocation (Nadler *et al*., 2010; Newton *et al*., 2010; Vossenkamper *et al*., 2010). This resulted in diminished IL-8 secretion in response to natural infection and stimulation with TNF. However, whereas NleE prevented both TNF- and IL-1β-stimulated IκB degradation, NleB inhibited the TNF pathway only suggesting that NleB and NleE have complementary but independent activities (Newton *et al*., 2010). In this study, we screened further effectors with unknown function for their ability to inhibit the translocation of p65 to the cell nucleus. During this screen, we observed that NleC induced degradation of p65 and other NF-κB signalling components. We suggest that NleC is a type III effector protease that acts synergistically with NleE and NleB to suppress immune activation.

## Results

### NleC induces degradation of the p65 subunit of NF-κB

**Fig. 1:**
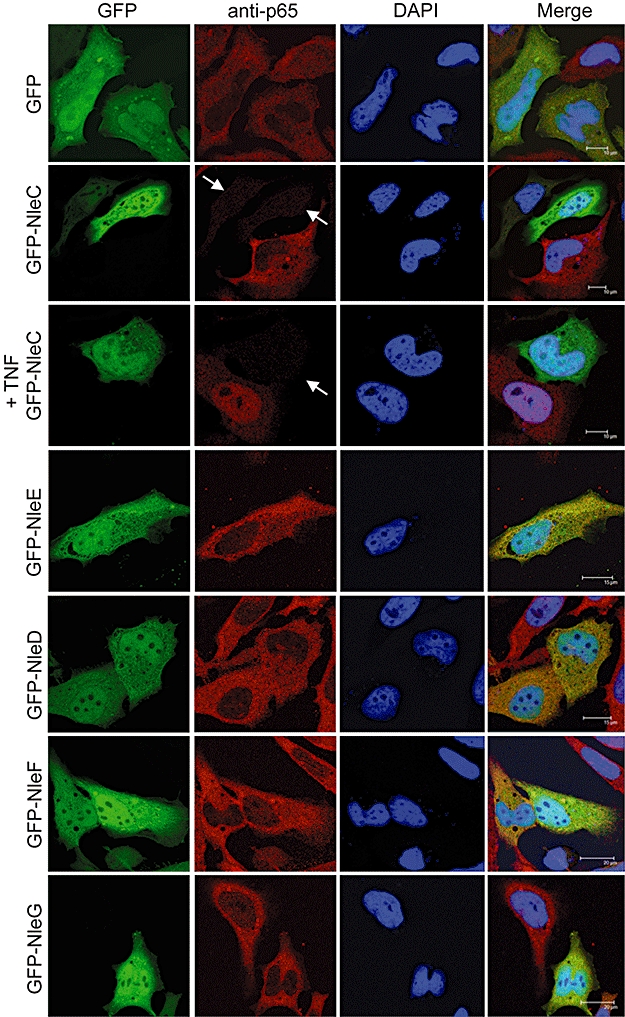
Effect of ectopically expressed NleC on p65 immunostaining. Representative immunofluorescence fields of p65 degradation using anti-p65 (red) in HeLa cells transfected with pEGFP–C2 (GFP), pGFP–NleC (GFP–NleC), pGFP–NleE, pGFP–NleD, pGFP–NleF and pGFP–NleG (green) and left unstimulated or stimulated with TNF for 30 min where indicated. Cell nuclei were stained with DAPI (blue). Transfections and staining were performed independently at least three times per GFP–effector fusion. Arrows indicate transfected cells with poor p65 staining.

In this study we screened further T3SS effectors from EPEC E2348/69 with no known function for their ability to inhibit the translocation of p65 to the cell nucleus in response to TNF or IL-1β using anti-p65 antibodies to stain cells expressing GFP–effector fusions ectopically. We observed that cells expressing GFP–NleC showed significantly weaker p65 staining than cells expressing GFP, GFP–NleE, GFP–NleD, GFP–NleF, GFP–NleG, suggesting that p65 was undergoing degradation (Fig. 1). The weak p65 staining was independent of stimulation with TNF (Fig. 1).

**Fig. 2:**
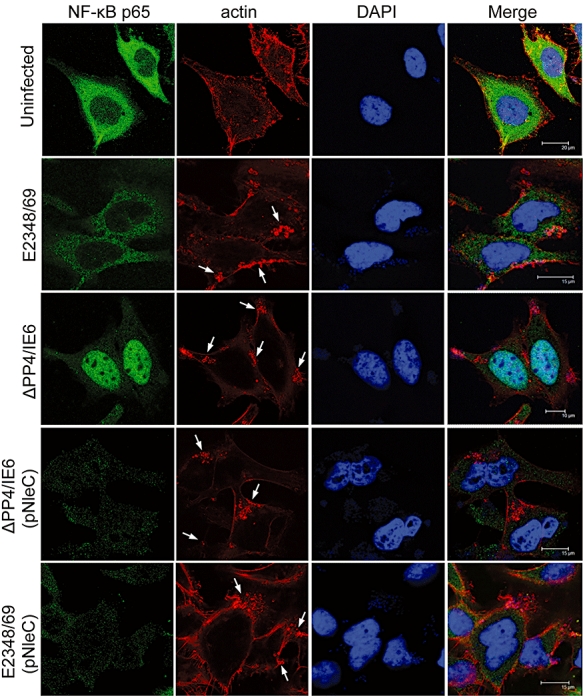
Effect of NleC on p65 during EPEC infection. Representative immunofluorescence fields showing p65 staining (green) in FAS-positive HeLa cells (red) uninfected or infected for 4 h with wild-type EPEC E2348/69, a ΔPP4/IE6 double island mutant, ΔPP4/IE6 carrying the overexpression vector pNleC or EPEC E2348/69 carrying the overexpression vector pNleC as indicated, and stained for nucleic acid with DAPI (blue). Infections and staining were performed independently at least three times per EPEC E2348/69 derivative. Arrows indicate FAS-positive lesions.

To investigate whether NleC delivered by the LEE-encoded T3SS could induce p65 degradation, we used derivatives of wild-type EPEC E2348/69 to infect HeLa cells. Successful infection was defined by a positive fluorescent actin staining (FAS) test, which served as a marker for the translocation of T3SS effectors. Although little p65 degradation was evident upon a 4 h infection with the wild-type strain EPEC E2348/69, the same wild-type strain expressing NleC from an IPTG-inducible promoter in pTrc99A (pNleC) induced clear degradation of p65 (Fig. 2). To avoid potential effects on NF-κB signalling from NleE and the two NleB homologues, NleB1 and NleB2, we also infected HeLa cells with a double island ΔPP4/IE6 mutant complemented with pNleC. The ΔPP4/IE6 mutant lacks genes encoding seven effectors including, NleE, NleB1, EspL, NleB2, NleC, NleD and NleG (Newton *et al*., 2010). Whereas the ΔPP4/IE6 mutant had no effect on levels of p65, ΔPP4/IE6 carrying pNleC induced p65 degradation similar to E2348/69 (pNleC) (Fig. 2).

### NleC is a putative zinc metalloprotease

**Fig. 3:**
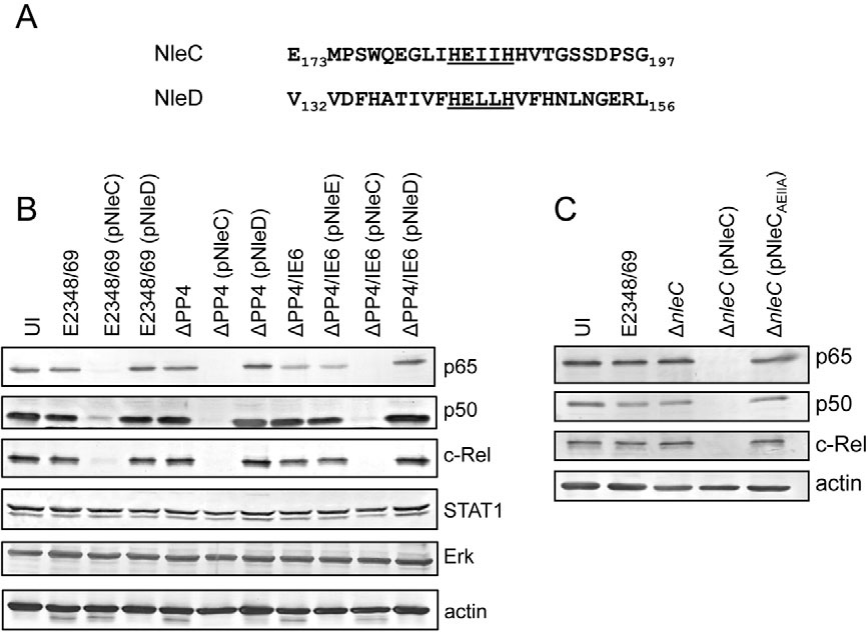
Effect of NleC on inflammatory signalling mediators.A. Alignment of the regions of NleC and NleD containing the putative zinc metalloprotease motif (underlined).B and C. Representative immunoblots showing degradation of inflammatory signalling mediators in HeLa cells infected with derivatives of EPEC E2348/69 for 4 h. Cells were harvested for immunoblotting and host proteins were detected with antibodies to p65, p50, c-Rel, STAT1 and Erk. Infections and immunoblotting were performed independently at least three times per EPEC E2348/69 derivative. Antibodies to actin were used as loading control. UI, uninfected.

The effect of NleC on p65 suggested that NleC may have protease activity. Analysis of the NleC amino acid sequence using Prosite (Sigrist *et al*., 2010) identified a putative consensus zinc metalloprotease active site, HEIIH (Fig. 3A). We also previously described a putative consensus zinc metalloprotease site, HELLH, in the downstream effector, NleD (Fig. 3A) (Marches *et al*., 2005). Both histidines within the consensus sequence of zinc metalloproteases (HExxH) are involved in binding a zinc ion that promotes nucleophilic attack on peptide bonds using a water molecule at the active site (Potempa and Pike, 2005).

To confirm the degradation of p65 by NleC, we infected HeLa cells with derivatives of EPEC E2348/69 overexpressing NleC, NleD or NleE and performed an immunoblot with antibodies raised to the N-terminus of p65. NleD and NleE were useful controls as NleD has no effect on NF-κB activation but possesses a putative protease active site while NleE inhibits NF-κB activation but has no consensus protease site (Newton *et al*., 2010). In cells infected with any of EPEC E2348/69 (pNleC), ΔPP4/IE6 (pNleC) or ΔPP4 (pNleC), the band corresponding to p65 was significantly reduced (Fig. 3B). None of the other E2348/69 derivatives had any effect on levels of p65. We also examined the effect of NleC on other NF-κB members (c-Rel and p50) and other host transcription factors, STAT1 and Erk. Overexpression of NleC also induced the degradation of c-Rel, and p50, but not STAT1, Erk, actin or calnexin (Fig. 3B and data not shown).

**Fig. 4:**
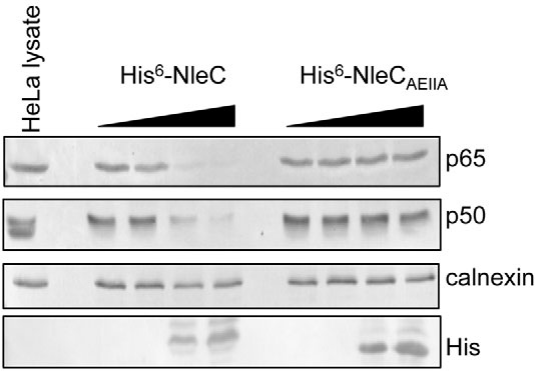
Effect of recombinant NleC on the degradation of NF-κB. Representative immunoblot showing degradation of Rel proteins in HeLa cells incubated with increasing concentrations of recombinant His^6^NleC and His^6^NleC_AEIIA_ (0.01, 0.1, 0.5 and 1 µg) for 4 h. Incubations were performed independently at least three times per His^6^NleC derivative. Cells were harvested for immunoblotting and Rel proteins were detected with antibodies to p65 and p50. Antibodies to calnexin were used as loading control for HeLa lysates. His^6^-tagged proteins were detected with anti-His antibodies.

Amino acid substitutions of the histidine residues in the active site of zinc metalloproteases render the enzymes non-functional (Jongeneel *et al*., 1989). Here we substituted His183 and His187 with alanine to test the effect of the consensus sequence on p65 degradation. The inactive form of NleC, NleC_AEIIA_, was then used to complement an *nleC* mutant of EPEC (Marches *et al*., 2005). While NleC expressed from pTrc99A induced degradation of p65, p50 and c-Rel, NleC_AEIIA_ was unable to degrade the same Rel proteins (Fig. 3C). To determine if NleC directly cleaved p65 and p50, we generated His^6^-NleC and His^6^-NleC_AEIIA_ and incubated the recombinant proteins with HeLa lysates. Levels of p65 and p50 were detected by immunoblot using antibodies to p65 and p50. With increasing concentrations of His^6^-NleC but not His^6^-NleC_AEIIA_, we observed increasing degradation of p65 and p50 suggesting that the metalloprotease acts directly on these substrates (Fig. 4). We assume the altered migration of His^6^-NleC_AEIIA_ (Fig. 4) was due to a change in the charge of the mutant protein arising from the substitution of two positively charged histidine residues with non-polar alanine residues.

### NleC acts synergistically with NleE

**Fig. 5:**
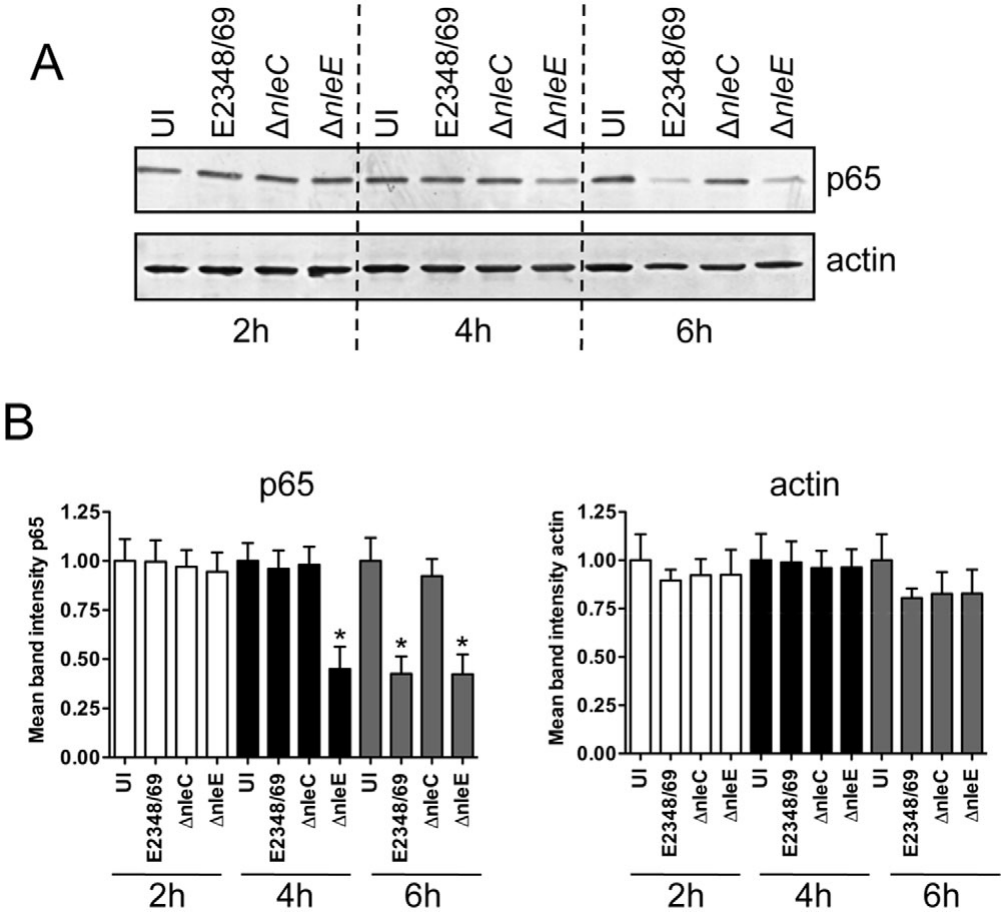
Effect of EPEC infection on p65 degradation.A. Immunoblot showing degradation of p65 in HeLa cells infected with derivatives of EPEC E2348/69 for 2, 4 and 6 h. Infections and immunoblotting were performed independently at least three times per EPEC E2348/69 derivative and per time point. Cells were harvested for immunoblotting and detected with antibodies to p65. Antibodies to actin were used as loading control. UI, uninfected.B. Mean band intensity of immunoblots performed on HeLa cells infected with derivatives of EPEC E2348/69 and detected with antibodies to p65 and actin as indicated. UI, uninfected. Results are expressed as the mean ± SEM of three independent experiments. *P < 0.05 compared with uninfected, unstimulated cells at each time point, one-way anova.

A remaining question in the proposed activity of NleC was the lack of obvious p65 degradation by wild-type EPEC E2348/69 during infection (Fig. 3B). This appeared to relate to the amount of NleC injected by the T3SS since overexpression of NleC in the wild-type background induced a significant reduction in levels of p65 (Fig. 3B). We postulated that NleC may preferentially target free p65/p50 dimer released from the IκB complex. If this were the case, p65 degradation by wild-type EPEC should be evident after longer infection times and in the absence of NleE, which inhibits IκB degradation. To test this hypothesis we performed 2, 4 and 6 h infections with EPEC E2348/69 and isogenic *nleE* and *nleC* mutants. While no p65 degradation was evident in cells infected with wild-type EPEC for 2 and 4 h (Fig. 5 and Newton *et al*., 2010), wild-type infection induced significant p65 degradation after 6 h (Fig. 5). The effect of NleC at 6 h may in part arise from increased numbers following bacterial replication and/or an accumulation of NleC over time. However, in the absence of *nleE*, where IκB is degraded and p65/p50 dimer is released, reduced levels of p65 could be observed 4 h after infection (Fig. 5). Thus, native levels of NleC delivered by EPEC do induce p65 degradation but this was only apparent in HeLa cells 6 h after infection or at 4 h in the absence of NleE.

**Fig. 6:**
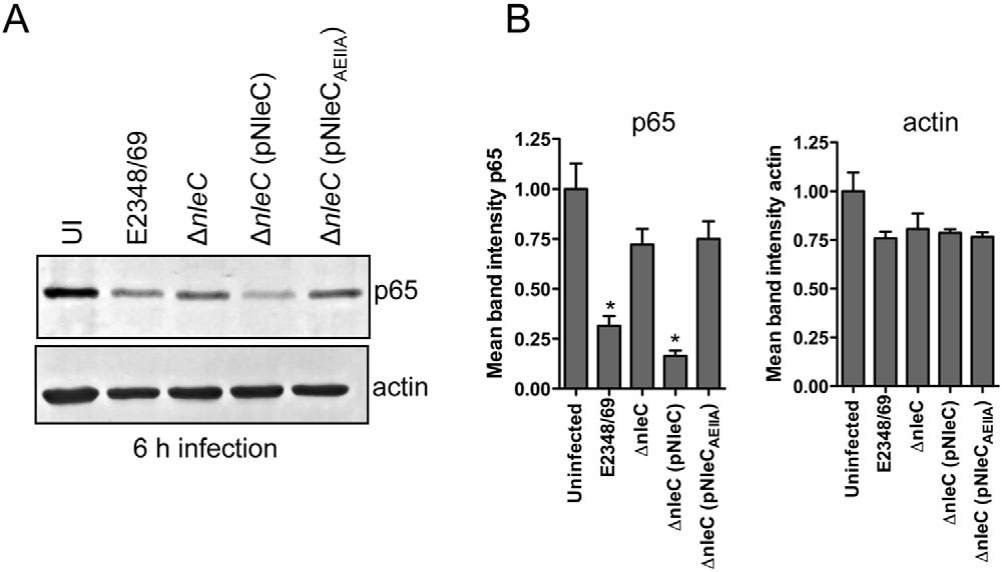
Effect of prolonged EPEC infection on p65 degradation.A. Immunoblot showing degradation of p65 in HeLa cells infected with derivatives of EPEC E2348/69 for 6 h. Infections and immunoblotting were performed independently at least three times per EPEC E2348/69 derivative. Cells were harvested for immunoblotting and detected with antibodies to p65. Antibodies to actin were used as loading control. UI, uninfected.B. Mean band intensity of immunoblots performed on HeLa cells infected with derivatives of EPEC E2348/69 and detected with antibodies to p65 and actin as indicated. Results are expressed as the mean ± SEM of three independent experiments. *P < 0.05 compared with uninfected, unstimulated cells at each time point, one-way anova.

To confirm that NleC was responsible for the degradation of p65 at this later time point, we infected cells with the *nleC* mutant complemented with pNleC or pNleC_AEIIA_. NleC expressed from pTrc99A induced degradation of p65 whereas NleC_AEIIA_ lacked this activity (Fig. 6). There is also some suggestion of cell loss at this prolonged time point as all infected samples exhibited a trend towards reduced levels of actin, although this was not significant compared with the uninfected control sample (Figs 5 and 6).

### NleC inhibits IL-8 secretion

**Fig. 7:**
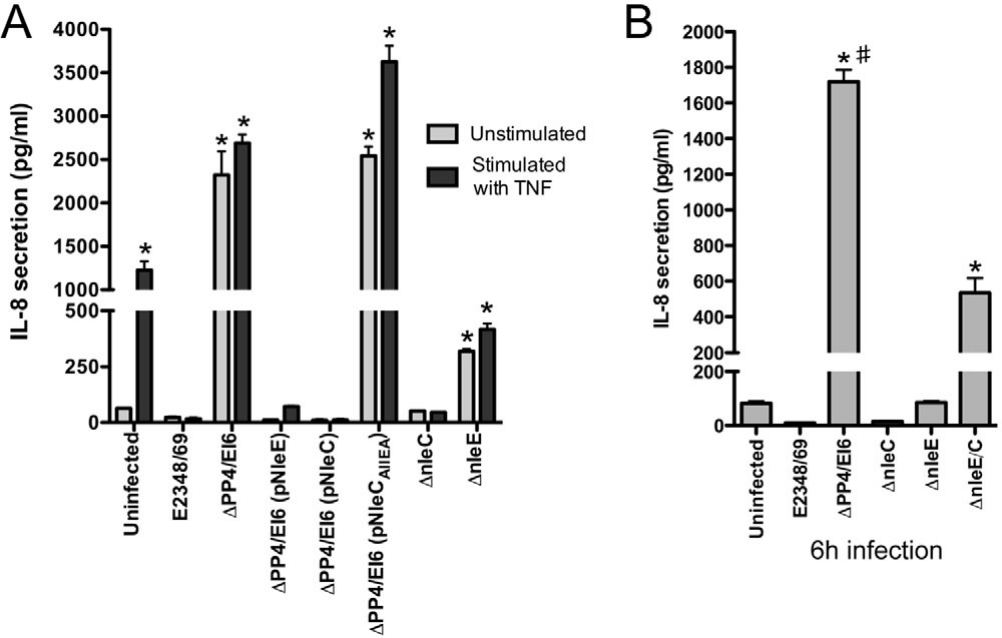
Effect of *nleE* and *nleC* mutations on IL-8 secretion from infected HeLa cells.A. HeLa cells were infected with derivatives of EPEC E2348/69 for 4 h and left unstimulated (light grey bars) or stimulated with TNF for 24 h (dark grey bars). Results are the mean ± SEM of at least three independent experiments carried out in duplicate. *P < 0.0001 compared with uninfected, unstimulated cells, one-way anova.B. HeLa cells were infected with derivatives of EPEC E2348/69 for 6 h and left unstimulated (light grey bars). Results are the mean ± SEM of at least three independent experiments carried out in duplicate. *P < 0.0001 compared with uninfected, unstimulated cells, one-way anova. #ΔPP4/IE6 significantly greater than *nleE/C* double mutant *P* < 0.0001, unpaired two-tailed *t*-test.

We previously showed that an *nleE* mutant of EPEC had a reduced capacity to inhibit IL-8 expression and secretion during infection, although a T3SS system mutant was even further impaired (Newton *et al*., 2010). Here we found that the double PP4/IE6 island mutant which lacks both *nleE* and *nleC* was unable to inhibit IL-8 secretion, similar to a T3SS mutant (Fig. 7 and data not shown). Complementation of PP4/IE6 with either pNleE or pNleC but not pNleC_AEIIA_ led to the inhibition of IL-8 secretion similar to wild-type EPEC indicating that the effectors have comparable functions (Fig. 7A). The *nleE* mutant showed an impaired ability to inhibit IL-8 secretion 4 h after infection but the level of IL-8 was still significantly lower than that induced by the ΔPP4/IE6 double island mutant, which also lacks *nleC* (Fig. 7A). Interestingly, 4 h after infection, an *nleC* mutant inhibited IL-8 secretion to the same degree as wild-type EPEC (Fig. 7A), suggesting that NleE delivered by the *nleC* mutant was sufficient for the suppression of immune activation at this time point. Stimulation with TNF made little difference to the ability of EPEC to inhibit IL-8 secretion (Fig. 7A).

To determine if NleC played a role in the inhibition of IL-8 secretion, we examined the effect of prolonged EPEC infection on IL-8 levels where we had previously seen evidence of p65 degradation by wild-type EPEC due to NleC activity (Figs 5 and 6). We compared IL-8 secretion from cells infected for 6 h with wild-type EPEC, the ΔPP4/IE6 double island mutant, the single Δ*nleE* and Δ*nleC* mutants and a double Δ*nleE/C* mutant. At this time point, the Δ*nleC* mutant still inhibited IL-8 secretion to wild-type levels presumably due to NleE activity (Fig. 7B). In addition, at this time point there was now no significant difference in IL-8 secretion between the *nleE* mutant and wild-type EPEC. In contrast, a double *nleE/C* mutant was significantly impaired in its ability to inhibit IL-8 secretion 6 h after infection, suggesting that NleE and NleC act synergistically (Fig. 7B). However, IL-8 secretion resulting from infection with the ΔPP4/IE6 double island mutant was still significantly greater than that induced by the double *nleE/C* mutant indicating that further effectors encoded within PP4 and/or IE6 may have an anti-inflammatory role (Fig. 7B)

## Discussion

Enteropathogenic *E. coli* and EHEC stimulate inflammatory signalling through TLR5 recognition of flagellin (Miyamoto *et al*., 2006; Badea *et al*., 2009; Schuller *et al*., 2009). *Salmonella* also stimulates TLR signalling (Tapping *et al*., 2000; Hayashi *et al*., 2001) and, similar to *Shigella*, activates intracellular NLRs that recognize bacterial ligands such as flagellin, peptidoglycan and the T3SS itself (Miao *et al*., 2006; 2010; Nigro *et al*., 2008). Intestinal inflammation contributes to disease pathology but is also required for pathogen control and clearance. Hence many bacterial enteropathogens have acquired effectors that modulate the inflammatory response as a way of persisting in the host. *Salmonella* and *Shigella* secrete multiple T3SS effectors that interfere with inflammatory signalling and suppress cytokine production. For example, OspF from *Shigella* and SpvC from *Salmonella* are phosphothreonine lyases that target mitogen-activated protein kinases (MAPKs) in the nucleus (Arbibe *et al*., 2007; Kramer *et al*., 2007; Li *et al*., 2007). OspG from *Shigella* is a protein kinase that binds ubiquitin-conjugating enzymes and inhibits the ubiquitination and degradation of phosphorylated IκB (Kim *et al*., 2005). In addition, IpaH_9.8_ from *Shigella* and its homologue SspH1 from *Salmonella* traffic to the nucleus where they act as E3 ubiquitin ligases that target selected signalling kinases for proteosome-dependent degradation (Haraga and Miller, 2006; Rohde *et al*., 2007; Ashida *et al*., 2010). The activity of OspF, OspG, SpvC, IpaH_9.8_ and SspH 1 results in the repression of NF-κB-dependent genes such as *IL8* leading to immune suppression post bacterial invasion.

Recently, we and Nadler *et al*. found that NleE/OspZ, an effector shared by A/E pathogens and *Shigella*, and NleB, inhibited the degradation of IκB leading to reduced IL-8 production (Nadler *et al*., 2010; Newton *et al*., 2010; Vossenkamper *et al*., 2010). Although the mechanism of action of NleE and NleB is still under investigation, here we continued screening other type III effectors for their ability to inhibit activation of inflammatory signalling. Our approach was to express the effectors ectopically by transfection as GFP-fusion proteins. Through this screening we found that ectopic expression of GFP–NleC resulted in degradation of the NF-κB subunit, p65. p65 degradation was also evident upon translocation of NleC by the T3SS of EPEC. Other Rel proteins, p50 and c-Rel, were also affected by NleC but not the transcription factors, STAT1 and Erk. Degradation of the Rel proteins required the zinc metalloprotease motif of NleC, H_183_EIIH, and recombinant His^6^-NleC degraded p65 and p50 in HeLa cell lysates suggesting direct cleavage by the protease effector. Indeed, while this work was under review, two independent studies demonstrated direct cleavage of recombinant p65 by NleC (Yen *et al*., 2010; Baruch *et al*., 2011). Both studies also showed that p65 cleavage occurred at the N-terminus in the Rel homology domain and that cleavage was independent of the cell proteasome. A third study confirmed proteasome-independent cleavage and suggested that NleC also targets IκB (Muehlen *et al*., 2011).

In this study, we observed p65 degradation by wild-type EPEC translocating native levels of NleC only after prolonged infection whereas p65 degradation was evident earlier following infection with an *nleE* mutant. Although further work is required to establish clearly the hierarchy of T3SS effector activity, we are investigating a model whereby NleE acts early to prevent IκB degradation and p65/p50 release, while NleC acts later to degrade unbound p65/p50 before cytokine gene transcription is initiated in the cell nucleus. In support of this model, two recent studies showed that NleC has activity in the cell nucleus (Yen *et al*., 2010; Baruch *et al*., 2011). Therefore the temporal and spatial regulation of NleC and NleE activity may occur at several levels, namely gene transcription, effector translocation and/or post-translocation effector trafficking, which has been reported recently for EPEC PDZ-binding effectors (Martinez *et al*., 2010).

NleC and NleE mediated inhibition of NF-κB activation was mirrored in suppression of IL-8 secretion. IL-8 is an NF-κB responsive gene and is a physiological readout of NF-κB activity. Although *nleC* and *nleE* single mutants showed wild-type levels of IL-8 inhibition following prolonged infection, an *nleE/C* double mutant was significantly impaired for inhibition of IL-8 secretion. However, this deficiency was not as great as the double island mutant ΔPP4/IE6, suggesting that further effectors encoded with in these genomic islands, such as NleD which degrades the signalling kinase, JNK (Baruch *et al*., 2011), contribute to the ability of EPEC to inhibit inflammatory signalling. The apparent redundancy in function between NleE and NleC may also explain why previous reports have failed to find a strong phenotype associated with either an *nleE* or an *nleC* mutant during *Citrobacter rodentium* infection of mice (Marches *et al*., 2005; Kelly *et al*., 2006; Wickham *et al*., 2007a).

In addition to NleC from A/E pathogens, we detected NleC homologues in diverse non-*E. coli* pathogens by blastp analysis, including *Salmonella enterica* ssp. Enterica, *Arsenophonus nasoniae*, a pathogen of wasps and the aquatic pathogens, *Yersinia aldovae* and *Photobacterium damselae* ssp. Piscicida, all which have a T3SS (do Vale *et al*., 2005; Switt *et al*., 2009; Chen *et al*., 2010; Wilkes *et al*., 2010). The NleC homologue from *P. damselae* has been described previously as AIP56 for apoptosis-inducing protein 56 and this effector is required for the induction of apoptosis in phagocytes from sea bass (do Vale *et al*., 2005). Although amino acid similarity between NleC and AIP56 is only 58%, the metalloprotease active site is present and it is tempting to speculate that AIP56 targets NF-κB-like proteins for degradation, thereby inducing apoptosis (do Vale *et al*., 2005). Indeed NleC is not the first T3SS effector reported to target NF-κB. The tail-specific protease, CT441, from *Chlamydia trachomatis* is a T3SS protease that cleaves human p65 during infection of epithelial cells thereby inhibiting the host inflammatory response (Lad *et al*., 2007). For EPEC, the combined effects of NleE and NleC appear to allow the pathogen to exert exquisite control of NF-κB signalling stimulated by both TLR and death receptor signalling, and despite the fact that inflammation ultimately results from infection with EPEC, these effectors may allow the pathogen to delay innate immune responses long enough to establish infection and disseminate to other hosts (Wickham *et al*., 2007b).

## Experimental procedures

### Bacterial strains and growth conditions

The bacterial strains and plasmids used in this study are listed in Table S1. Bacteria were grown at 37°C in Luria–Bertani (LB) medium or Dulbecco's modified Eagle's (DMEM) where indicated and supplemented with ampicillin (100 µg ml^−1^), kanamycin (100 µg ml^−1^) or chloramphenicol (25 µg ml^−1^) when necessary.

### HeLa cell infection and IL-8 secretion

HeLa cells were cultured in T75 cm^2^ tissue culture flasks (Triple Red, UK) in DMEM supplemented with 10% FBS and 5% HEPES, 100 U ml^−1^ penicillin and 100 µg ml^−1^ streptomycin in 5% CO_2_ at 37°C. Sixteen to 24 h before either transfection or infection, cells were seeded onto 12 mm glass coverslips (Menzel-Glaser, Braunschweig, Germany) within 24-well tissue culture trays (Greiner Bio-One, Germany) at a density of 10^5^ cells per well. For infection, EPEC derivatives were grown in Luria broth (LB) for 8 h before being subcultured into DMEM and incubated stationary for approximately 16 h at 37°C with 5% CO_2_. Ten microlitres of this culture, OD_600_ = 1.0 nm, was used to infect each well for 2 h, 4 h or 6 h as indicated (∼5 × 10^6^ cfu). For analysis of IL-8 secretion, monolayers were infected for 4 h before being incubated for 8–12 h in media supplemented with 50 µg ml^−1^ gentamicin with or without 20 ng ml^−1^ TNF (Calbiochem, EMD4Biosciences, USA). IL-8 secretion into cell culture supernatants was measured by ELISA according to the manufacturer's instructions (R&D Systems, Minneapolis, MN). Differences in IL-8 secretion were assessed for significance by one-way analysis of variance (anova) with Tukey's Multiple Comparison post-test.

### DNA cloning, purification and sequence analysis

DNA-modifying enzymes were used in accordance with the manufacturer's recommendations (Roche). PCR amplification consisted of an initial denaturation step at 94°C for 2 min, followed by 30 cycles of 44 s at 94°C, 40 s at 40°C and 1 min at 70°C. PCR products and restriction digests were purified using the Wizard® SV Gel and PCR Clean-Up System (Promega, WI). The *nleC* gene of EPEC E2348/69 was amplified from genomic DNA by PCR using the primer pair NleC_F_/NleC_R_ (Table S2). The PCR product was digested with EcoRI/BamHI and ligated into pEGFP–C2 to generate an N-terminal GFP fusion to NleC (pGFP–NleC). pGFP–NleF and pGFP–NleG were constructed using an identical strategy to pGFP–NleC with EPEC E2348/69 as a template and the primer pairs NleF_F_/NleF_R_, NleG_F_/NleG_R_ respectively. To generate the complementing vector, pNleC, *nleC* from pGFP–NleC was digested with EcoRI and BamHI and ligated into pTrc99A. To generate the complementing vector, pNleD, *nleD* from EPEC E2348/69 was amplified from genomic DNA by PCR using the primer pair NleD_F_/NleD_R_ (Table S2). pNleC_AEIIA_ was created using the Stratagene Quickchange II Site-Directed Mutagenesis Kit. pNleC was used as template DNA and amplified by PCR using the primer pair pNleC_(AEIIA)F_/pNleC_(AEIIA)R_ (Table S2). N-terminal His^6^ tagged versions of NleC and NleC_AEIIA_ were generated by amplifying DNA from pEGFP–NleC or pEGFP–NleC_AEIIA_ by PCR using the primer pair NleC_F_/NleC_R_. The products were cloned into the EcoRI/SalI restriction sites of pET28a.

### Preparation of His^6^-NleC and His^6^-NleC_AEIIA_ and protease activity in HeLa lysates

Overnight cultures of BL21 (pET-NleC) and BL21 (pET-NleC_AEIIA_) grown in LB were diluted 1:100 in 200 ml of LB supplemented with kanamycin (100 µg ml^−1^) with shaking to an optical density of (*A*_600_) 0.6 at 37°C. Cells were induced with 1 mM IPTG and grown for a further 2 h then pelleted by centrifugation. Proteins were purified by nickel affinity chromatography in accordance with the manufacturer's instructions (Novagen, EMD4Biosciences, USA). Elution fractions had a total protein content of 1 mg ml^−1^.

HeLa cells were cultured for 48 h in 100 mm tissue culture dishes (Greiner Bio-One, Germany) in DMEM supplemented with 10% FBS and 5% HEPES, 100 U ml^−1^ penicillin and 100 µg ml^−1^ streptomycin in 5% CO_2_ at 37°C. Cells were collected in 600 µl of PBS using a cell scraper to detach the adherent monolayer followed by passage 30 times through a 22-inch needle for lysis. The lysate was kept on ice for 10 min before being transferred to a microfuge tube and centrifuged for 10 min at 4°C. The supernatant was removed to a separate tube and 100 µl aliquots were incubated at 37°C with varying amounts of His_6_-NleC or His_6_-NleC_AEIIA_ ranging from 0.01 µg to 1 µg for a period of 4 h. Degradation of cellular p65 and/or p50 was determined by immunoblot. Sample buffer was added to each tube, boiled and 15 µl was loaded onto 10% SDS-PAGE gels. Proteins were transferred to nitrocellulose membranes and probed with either rabbit polyclonal anti-p50 (Cell Signaling), rabbit polyclonal anti-p65 (Santa Cruz) or rabbit polyclonal anti-calnexin (Santa Cruz) diluted 1:1000 in TBS with 5% BSA (Sigma) and 0.1% Tween (Amresco). Proteins were detected using anti-rabbit IgG alkaline phosphatise-conjugated secondary antibodies (Sigma) diluted 1:5000 in TBS with 5% BSA and 0.1% Tween and developed with NBT/BCIP substrate (Thermo Scientific, Rockford, IL).

### Construction of EPEC E2348/69 *nleC/nleE* double mutant

The *nleC* gene was disrupted using the λ Red recombinase system in an EPEC E2348/69 Δ*nleE* background. Briefly, the kanamycin-resistance gene was amplified from pKD4 using the primers ΔnleC_F_ and ΔnleC_R_ (Table S2). PCR products were DpnI digested before being electroporated into EPEC E2348/69 Δ*nleE* carrying the Red recombinase expression plasmid, pKD46. Mutants were selected from LB plates supplemented with chloramphenicol and kanamycin and verified by PCR and sequencing for the replacement of *nleC* with the kanamycin-resistance gene.

### Immunofluorescence, fluorescence actin staining test and confocal microscopy

HeLa cells were transfected with plasmids using Lipofectamine 2000 (Invitrogen, Carlsbad CA, USA) according to the manufacturer's specifications. Transfected HeLa cells were left unstimulated or stimulated with 20 ng ml^−1^ TNF (Calbiochem) for 30 min at 37°C and 5% CO_2_. Transfected or infected cells were fixed with 3.7% (w/v) formaldehyde (Sigma) in PBS for 10 min and permeabilized with 1:1 (v/v) methanol-acetone at −20°C for 15 min. Cells were then blocked in PBS with 3% (w/v) BSA (Sigma) for 30 min. For visualization of NF-κB p65 in transfected cells, a rabbit polyclonal anti-p65 (SC-109, Santa Cruz, CA, USA) antibody was applied at 1:100 in PBS with 3% (w/v) BSA for 1 h at 20°C. Alexa Fluor 488 or Alexa Fluor 568 (Invitrogen) conjugated anti-rabbit immunoglobulin G was used as secondary antibodies at 1:2000 for 1 h at 20°C. 4′,6-Diamidino-2-phenylindole (DAPI; Invitrogen) was applied at a dilution of 1:20000 in blocking solution for 5 min at room temperature post secondary antibody treatment and coverslips were mounted on glass slides with Fluorescence Mounting Medium (Dako, Carpinteria, CA). For infected HeLa cells, the fluorescence actin staining (FAS) test was achieved by fixing and permeabilizing as described above and including 0.5 mg ml^−1^ phalloidin conjugated to rhodamine (Sigma) during the primary antibody incubation. Images were acquired using a confocal laser scanning microscope (Leica LCS SP2 confocal imaging system) with a 100×/1.4 NA HCX PL APO CS oil immersion objective.

### Detection of Rel proteins by immunoblot

To test the effect of NleC on host signalling factors, HeLa cells were infected with derivatives of EPEC E2348/69 for 2, 4 or 6 h. Cells were lysed with 60 µl of lysis buffer (50 mM Tris-HCl pH 7.4, 1 mM EDTA, 150 mM NaCl, 1% Triton X-100, 2 mM Na_3_VO_4_, 10 mM NaF, 1 mM PMSF, Roche Complete Protease Inhibitor EDTA-free) by incubation on ice for 5 min before the lysate was transferred to a microfuge tube and incubated on ice for a further 10 min. The lysate was centrifuged for 10 min at 4°C and equal volumes of supernatant were removed to a tube containing sample buffer, boiled for 5 min before loaded onto 10% SDS-PAGE gels. Proteins were transferred to nitrocellulose membranes and probed with one of the following antibodies: rabbit polyclonal anti-c-Rel, rabbit polyclonal anti-p50, mouse monoclonal anti-Stat1 9H2 (Cell Signaling), rabbit polyclonal anti-p65 (Santa Cruz), mouse monoclonal anti-β-actin AC-15 (Sigma), mouse monoclonal anti-ERK clone 16 (BD Biosciences) or rabbit polyclonal anti-Calnexin (Stressgen). Actin or calnexin was used as loading controls. All antibodies were diluted 1:1000 in TBS with 5% BSA (Sigma) and 0.1% Tween (Amresco). Proteins were detected using anti-rabbit or mouse IgG alkaline phosphatise-conjugated secondary antibodies (Sigma) and developed with NBT/BCIP substrate (Thermo Scientific, Rockford, IL). All secondary antibodies were diluted 1:5000 in TBS with 5% BSA (Sigma) and 0.1% Tween (Amresco).

For quantification of band intensity, proteins were detected using peroxidase-conjugated secondary antibodies and developed with SuperSignal® West Pico Chemiluminescent Substrate (Thermo Scientific). Images were visualized using a Kodak Image Station 4000MM (IS4000MM) and processed using Carestream Molecular Imaging Software v5.0.2. Mean intensity data for bands were acquired by selecting regions of interest and subtracting background membrane intensity. For each time point values were normalized to protein levels of uninfected cells at that time point. Differences in band intensity were assessed for significance by one-way anova with Tukey's Multiple Comparison post-test.
