# Supplementary material for: A type III effector protease NleC from enteropathogenic Escherichia coli targets NF-κB for degradation
Source: Mol Microbiol. 2011 Feb 22;80(1):219–30. doi: 10.1111/j.1365-2958.2011.07568.x (PMC3178796; doi:10.1111/j.1365-2958.2011.07568.x)
Supplement: Supplementary file 1 [file mmi0080-0219-SD1.pdf]

SUPPLEMENTARY MATERIAL

Table S1. Bacterial strains and plasmids used in this study.

Strains	Characteristics	Source/reference
EPEC E2348/69	Wild type EPEC O127:H6	(Levine *et al.* 1978)
Δ*nleC*	EPEC E2348/69 Δ*nleC* Kan^R	(Marches *et al.* 2005)
Δ*nleE*	EPEC E2348/69 Δ*nleE* Cm^R	(Zurawski *et al.* 2008)
ΔPP4	EPEC E2348/69 PP4 island 1 deletion	(Vossenkamper 2010)
ΔPP4/IE6	EPEC E2348/69 PP4/IE6 double island 1 and 4 deletion	(Vossenkamper 2010)
Δ*escN*	EPEC E2348/69 Δ*escN* Kan^R	(Newton *et al.* 2010)
Δ*nleE/C*	EPEC E2348/69 Δ*nleE/nleC* Cm^R Kan^R	This study
**Plasmids**		
pTrc99A	Cloning vector for expression of proteins from P*trc*	Pharmacia Biotech
pNleC	*nleC* from EPEC E2348/69 in pTrc99A	This study
pNleD	*nleD* from EPEC E2348/69 in pTrc99A	This study
$pNleC_{AEIIA}$	*nleC* from EPEC E2348/69 carrying the mutations H183A and H187A in pTrc99A	This study
pEGFP-C2	Green fluorescent protein (GFP) expression vector	Clontech
pGFP-NleE	*nleE* from EPEC E2348/69 in pEGFP-C2	(Newton *et al.* 2010)
pGFP-NleC	*nleC* from EPEC E2348/69 in pEGFP-C2	This study
pGFP-NleF	*nleF* from EPEC E2348/69 in pEGFP-C2	This study
pGFP-NleG	*nleG* from EPEC E2348/69 in pEGFP-C2	This study
pGFP-NleD	*nleD* from EPEC E2348/69 in pEGFP-C2	(Newton *et al.* 2010)
pET28a	Expression vector for generation of His^6-tagged proteins	Novagen
pET-NleC	*nleC* from EPEC E2348/69 in pET28a	This study
$pET\text{-}NleC_{AEIIA}$	$nleC_{AEIIA}$ derived from $pNleC_{AEIIA}$ in pET28a	This study

SUPPLEMENTARY MATERIAL

Table S2. Oligonucleotide primers used in this study for vector construction.

Primer	Sequence 5'-3'
$NleC_F$	AA GAA TTC ATG AAA ATT CCC TCA TTA CAG
$NleC_R$	GCG GTG GAT CCC TCA TCG CTG ATT GTG TTT GTC
$pNleC_{(AEIIA)F}$	CG TGG CAG GAA GGA CTG ATT GCC GAG ATT ATT GCT CAT GTT ACT GGA TCT AGC
$pNleC_{(AEIIA)R}$	GCT AGA TCC AGT AAC ATG AGC AAT AAT CTC GGC AAT CAG TCC TTC CTG CCA CG
$NleF_F$	AA GAA TTC ATG TTA CCA ACA AGT GGT TCT
$NleF_R$	GCG GTG GAT CCC TCA TCC ACA TTG TAA AGA TCC
$NleG_F$	AA GAA TTC ATG CCA TCA TTA GTT TCA GG
$NleG_R$	GCG GTG GAT CCC TCA CTT ATC CTT TAT GAC AAA G
$NleD_F$	AA GAA TTC ATG CGC CCT ACG TCC CTC
$NleD_R$	GCG GTG GAT CCC CTA AAG CAA TGG ATG CAG TC
$NleE_F$	AA GAA TTC ATG ATT AAT CCT GTT ACT A
$NleE_R$	GGG ATC CGT CTA CTC AAT TTT AGA AAG
$\Delta nleC_F$	GAA GCA GCT CCA GCC TAC ACA GCC TGC TAC GGC TGT CAT GG
$\Delta nleC_R$	CTA AGG AGG ATA TTC ATA GCT CGC ACG TCG TGT CGC TC
